# Psychosocial Health Experiences and Coping Strategies of Family Caregivers of Adults With Cancer: A Qualitative Study

**DOI:** 10.1002/hsr2.71857

**Published:** 2026-02-22

**Authors:** Catherine Mensah, Salome Amissah‐Essel, Nancy Innocentia Ebu Enyan

**Affiliations:** ^1^ Tamale Teaching Hospital Tamale Ghana; ^2^ Department of Health, Physical Education and Recreation Faculty of Science and Technology Education, College of Education Studies, University of Cape Coast Cape Coast Ghana; ^3^ Department of Public Health Nursing School of Nursing and Midwifery, College of Health and Allied Sciences, University of Cape Coast Cape Coast Ghana

**Keywords:** adults, cancer, family caregivers, Ghana, psychosocial experiences

## Abstract

**Background and Aims:**

Family caregivers play an important, all‐encompassing role in supporting adult patients diagnosed with cancer. This study explored the psychosocial health experiences and coping strategies of family caregivers of adults with cancer in a Ghanaian Teaching Hospital.

**Methods:**

The study employed a descriptive exploratory qualitative design. Fourteen family caregivers of adult patients with cancer were purposively selected for the study. A semi‐structured interview guide was used to collect data, which was then analysed using thematic analysis.

**Results:**

The family caregivers were aged between 20 to 65 years. Twelve out of the 14 care recipients were females, and their ages ranged from 41 to 70 years, with six aged between 41 and 50 years. Nine themes emerged from the study and were grouped under the four main dimensions of psychosocial health. Mental health: “The thinking is many” and “I always feel depressed”; Emotional health: “Your whole mood changes” and “I am very frustrated”; Social Health: “I don't have time” and “Neglect”. Three themes were identified as coping strategies, of which one fell within the spiritual health dimension: “I know that my God lives” and two on social health: “We try to adjust” and “Family support”.

**Conclusion:**

Efforts to improve the quality of life of family caregivers of adult patients with cancer should focus on interventions that address the mental, emotional and social aspects of life. It is recommended that nurses consider the mental, emotional, social, spiritual and financial aspects of the family caregiving process as part of the effort to deliver comprehensive family‐centered care.

## 1 Introduction

Family caregivers [FCGs] play critical roles in the lives of people living with cancer. FCGs refer to individuals, either related or unrelated to the person living with cancer, who provide direct care and support [1]. They include blood relatives, spouses, friends and housekeepers who provide a broad range of continuous assistance by performing activities of daily living for cancer patients in the hospital, home, or community setting [2]. In some resource‐constrained countries, FCGs actively participate in the provision of care to hospitalized adult patients with cancer due to inadequate resources for cancer care, which tends to produce a FCG burden [3, 4]. Caring for an adult cancer patient is a challenging experience for the FCG [5, 6, 7].

The family caregiving process could affect the health of the caregiver and impact their physical, mental, and social well‐being [8, 9]. This is because FCGs of adult patients with cancer participate in the pathways of cancer diagnosis, treatment, survivorship, and end‐of‐life care [1]. These often result in a caregiver burden that usually leads to deterioration in the quality of life of the caregiver, as caregiving requires much time and effort, great physical and emotional energy expenditure, and also hinders the caregiver's social engagements. Litzelman, Green, and Yabroff [10] asserted that the role of caregiving can be highly stressful and burdensome, and subsequently, compromises the caregiver's quality of life, which could lead to considerable impairment of physical, psychological and social health.

Evidence suggests that FCGs continue to face psychological and social challenges in caring for adult patients with cancer, which is often due to the repeated actions they have to perform for the patients in times of need [9]. For instance, in a study regarding sick leave usage of spouses of adult patients with cancer, it was reported that spouses of lung cancer patients had the most sick leave episodes, which may relate to the higher physical and emotional burdens of care [11, 12]. This, when unresolved, could lead to loneliness, tiredness, depression, mood swings, stress, sleep disturbance, fatigue, and unhealthy behaviors for caregivers [9, 13, 14]. Other psychosocial health experiences cited in previous studies include anxiety, fear, psychosomatic symptoms, marital problems, and physical symptoms such as tiredness, anorexia, indigestion, constipation, serious sleeping disorders, and pain [9, 15, 16, 17].

Nonetheless, FCGs play varied roles in promoting recovery, rehabilitation and enabling patients with cancer to cope with the disease. The interval and quality of recovery are closely linked with the psychological burden, stress level and general well‐being of the FCG [7]. This highlights their need for information on health and rehabilitation, including emotional and social support, along with financial advice and possible assistance to equip them in taking care of the patient [4]. This study, however, was quantitative in nature and so could not explore the psychosocial experiences FCGs face. Additionally, many FCGs experience high financial burdens in the care of their patients because of the low coverage of the National Health Insurance Scheme (NHIS) in Ghana in the area of cancer care and the high cost of medical care. Cancer treatment is costly, and most cancer medications are not covered by the NHIS. This may pose a greater financial challenge for FCGs.

Despite the continued relevance of FCGs, there is a dearth of literature on this subject in Ghana. A study on caregiving experiences of FCGs conducted in Ghana among patients with advanced breast cancer showed that sociocultural obligation and reciprocity were the main reasons for assuming the caregiving role. Further, the study also highlighted that the caregiver provided multi‐dimensional forms of support, such as physical, psychosocial, emotional, financial, symptom management, and spiritual support for women living with advanced breast cancer. Financial burden through the provision of out‐of‐pocket money for treatment costs and other related non‐medical costs was the main challenge reported by participants [18]. In an earlier study, the caregiver's spirituality buffered the adverse effect of caregiving stress on mental and physical functioning [19].

The present study was underpinned by the biopsychosocial model of health [20]. This model attempts to render a holistic perception of health. The support FCGs provide to adults with cancer could be influenced by the FCGs’ biological, psychological and social attributes. This model was useful in explaining how the health of FCGs was affected psychologically and socially by the caregiving process. This study, therefore, explored the psychosocial health experiences and coping strategies of FCGs of adults with cancer in a Ghanaian teaching hospital.

## 2 Methods

### 2.1 Study Design and Setting

This study employed an exploratory, descriptive qualitative design to (1) examine the psychosocial health experiences of FCGs caring for adults with cancer in Tamale Teaching Hospital (TTH), and (2) examine the coping strategies used by FCGs caring for adults with cancer in TTH. According to Creswell [21], the exploratory descriptive research design is conducted to investigate a research problem that has not been clearly defined or extensively studied. The design is instrumental in understanding how the phenomenon works. This study was conducted in the TTH, which is located in the Eastern part of the Tamale Metropolis of Ghana and serves as a major referral facility for other neighboring regions in Ghana and beyond countries [22].

The study was performed in accordance with the ethical standards as laid down in the 1964 Declaration of Helsinki. The study was approved by the Ethical Review Committee of the Ghana Health Service with reference number (GHS/RDD/ERC/Admin/APP/21/279). The first author established rapport with the participants before the interviews and assured them of the confidentiality of all information. The participants understood participation was voluntary. The purpose of the study was explained to obtain written informed consent after recruitment. Participants were informed of their right to withdraw from the study at any time without facing any consequences. Participants had the autonomy to schedule the date, time and suitable place for the meeting. There were no monetary benefits associated with the study. However, participants were offered counseling in case of emotional breakdown at any time during an interview session, at no cost. A preprint of this paper can be found on an SSRN server (https://papers.ssrn.com/sol3/papers.cfm?abstract_id=4258728).

### 2.2 Population

FCGs of adult patients diagnosed with cancer at the TTH constituted the study population. It is estimated that 600 FCGs were caring for patients diagnosed with cancer at the time of the study [23]. FCGs of adult patients with cancer aged 18 years and above and willing to participate in the study were included in the study. Those excluded from this study were FCGs who were under 18 years of age, those brought in to support another person at the time of the data collection, and those who were not willing to participate in the study.

### 2.3 Sampling

Participants were selected using a maximum variation type of purposive sampling to capture the unique and diverse views of the participants [24]. Consistent with the adopted sampling approach, participants were selected based on the inclusion criteria. Sampled FCGs were approached and briefed about the research, and those who were interested and willing to participate were recruited. The participants provided informed, voluntary, written consent after all study procedures had been fully explained. Data saturation was reached on the 14th participant.

### 2.4 Data Collection Instrument and Procedure

A semi‐structured interview guide was developed and pretested on two FCGs, but the data were not included in the analysis. The guide covered broad questions based on the literature on family caregiving, as indicated in Table 1. These were followed by probing questions to elicit a deep understanding and explanation of the concepts. The first author, with her experience in qualitative data collection, established rapport with the participants before conducting the interviews. Participants were allowed to schedule a time to enable full participation in the process. The interviews were conducted at a quiet place within the hospital to avoid disruptions that could affect the quality of the data.

**Table 1:** Interview guide.

Interview questions
Q1. What were some of your psychological experiences as a caregiver to a relative diagnosed with cancer? Q2. Has the provision of care affected your social life? What were some of your social experiences? Q3. How do you cope with the pressures of caring for your loved one?

Face‐to‐face data collection was conducted at the hospital by the first author. The first author and the participants adhered to the coronavirus disease protocols throughout the interactions. In all, 14 FCGs were interviewed as the authors believed that theoretical data saturation had been attained. Guest et al. [25] noted that data saturation may be attained by as few as six interviews, depending on the sample size of the population. However, it may be best to think of data in terms of richness and thickness [26] rather than the size of the sample [27]. Based on the review of transcripts of each interview session, the authors concluded that theoretical data saturation had occurred after the 14th participant.

Each interview took approximately 1 h, and was conducted in either the English language, Dagbani or Twi, both local dialects in Ghana. All interviews were audio‐taped and transcribed verbatim. Those that were in the local dialects were translated into English. All the transcriptions were read and analysed independently by the three authors. Consensus regarding natural units, categories and subcategories during the analysis of the interviews was reached by discussion between the researchers.

### 2.5 Data Analysis

The data were analysed by all the authors using the six‐step approach to thematic analysis by Clarke and Braun [28]. This entailed: (1) Familiarization with the data by reading and re‐reading the whole data to be more familiar with it. (2) Initial codes were then generated from the data. (3) After all the data items had been coded, themes were generated by interpreting aggregated meanings across the data. (4) The potential themes were reviewed, (5) the themes were defined and named, and (6) a report was produced [28].

The data were transcribed by the first author, and the second and third authors confirmed the transcription. The research team familiarized and immersed themselves in the data and analysed concurrently by actively reading the 14 transcripts line‐by‐line multiple times, searching for patterns and meanings. This process allowed a familiarization with the overall content of the data. Handwritten notes were made independently, by categorizing concepts that emerged during the data analysis process. An initial list of themes was generated independently to meaningfully and systematically organize the data after the categorization process. Disagreements in assignments or descriptions of themes were resolved through discussion and consensus. The final list of themes was categorized, organized, and refined. Through this iterative process of refinement of the initial themes, subthemes, and a more in‐depth meaning emerged of the participants’ experiences. An inductive approach was adopted whereby themes were strongly linked to the data.

### 2.6 Trustworthiness

Guba and Lincoln's principles of credibility, dependability, confirmability and transferability were adhered to in this study [29]. In maintaining credibility, we ensured prolonged engagements with the participants. In doing so, the period stated for the data collection was adhered to as part of efforts to gather relevant data for the study. The first author familiarized herself with the study setting and participants before the data collection. The data were sent back to the participants for their feedback to confirm the accuracy of the information.

To ensure dependability, all relevant information about the steps of the study was explained, and the findings were clearly explained for easy reading. Also, to address the dependability issue in the study, the processes within the study were reported in detail to provide an opportunity for the work to be replicated.

The interviewer was a female, bachelor of science in nursing‐prepared nurse with experience in working with patients living with cancer. At the time of this study, she was pursuing a membership program in oncology nursing. She engaged in self‐reflection throughout the process to ensure that her own perspectives and biases were not introduced into the work. Additionally, a reflexive journal was kept to ensure that the interpretations of the data were not influenced by the researchers’ prejudices, knowledge, and experiences to achieve confirmability.

## 3 Results

The study sample involved 14 participants who were FCGs of patients at different stages of cancer. Table 2 shows that the participants were aged between 20 and 65 years. Fifty percent of the participants were married. The occupation of the caregivers was varied. It included formal workers, such as teachers, agriculture extension officers and informal workers, such as traders. Also, 10 participants had a tertiary level of education. The length of the participants’ experiences with the caregiver role ranged from 1 month to 8 years.

**Table 2:** Demographic data of participants.

Participant's ID	Age (years)	Marital status	Level of education	Occupation of participants	Experience with caregiving
P 1	33 years	Married	Tertiary	Electrician	7 years
P 2	36 years	Married	Tertiary	Teacher	6 years
P 3	22 years	Not married	Tertiary	Student	8 months
P 4	65 years	Married	Tertiary	Health educator	5 months
P 5	23 years	Not married	Tertiary	Teacher	4 years
P 6	46 years	Married	Junior high	Trader and farmer	1 year 4 months
P 7	31years	Married	Tertiary	Agric extension officer	1 month
P 8	27 years	Not married	Tertiary	Student	6 months
P 9	21years	Not married	Tertiary	Student	1 month
P 10	20 years	Not married	Senior high	Student	3 years
P 11	26 years	Not married	Tertiary	Trader	5 months
P 12	35 years	Married	Tertiary	Teacher	8 months
P 13	28 years	Not married	Senior high	Trader	8 years
P 14	42 years	Married	Basic	Trader	4 years

Table 3 indicates that 12 out of the 14 care recipients were females. Their ages ranged from 41 to 70 years, with six aged between 41 and 50 years. Ten of the care recipients were in Stages II and III of cancer. These stages are generally characterized by cancers that have grown more deeply into nearby tissues. They may have also spread to lymph nodes, but not to other parts of the body.

**Table 3:** Background characteristics of care recipients.

Background characteristic	Frequency
Gender	
Male	2
Female	12
Age	
41–50	6
51–60	5
61–70	3
Stage of cancer	
Stage I	2
Stages II and III	10
Stage IV	2

The most common themes that emerged from our analysis are presented below in line with the constructs being examined. It must be pointed out that the experiences of the participants did not differ regarding the stages of the cancer care recipient. As a result, the voices of the participants reveal the general experiences of FCGs.

### 3.1 Psychosocial Health Experiences of FCGs of Adult Patients With Cancer

Findings on the psychosocial health experiences were grouped under the four main dimensions of psychosocial health: mental, emotional, social, and spiritual health.

#### 3.1.1 Mental Health Experiences

Two themes emerged from participants’ mental health experiences as they care for their relatives with cancer. The themes are: “The thinking is many” and “I always feel depressed”.

*The thinking is that many*.

Participants indicated in their narratives that, in terms of mental health, caring for their relative with cancer set their minds thinking all the time, which sometimes leads to confusion. Some participants shared these:

“The thinking is many…you may be thinking that after treatment, your relative will be free and you will be okay, but they are saying that after the treatment, she may not live long” (P3)

“Every day, I think a lot, especially because of the things she has been saying. I've lost some weight too. I have headaches sometimes” (P5).

“I am confused because I don't know where the disease is coming from…” (P9)

“When we are learning, I will be thinking about my mother's sickness, and this has affected my studies” (P10).

*I always feel depressed*.

Participants also talked about how the caring experience affects their mental health in other ways. They narrated how they feel depressed and the stress they go through during the caring process. They shared these:

“I always feel depressed and sad each day I'm with her because of what they have been saying about cancer…” (P3)

“I felt there was no hope…people think that when you are suffering from this kind of sickness, then you are going to die like some people died. This kind of sickness has killed a lot of people. I know many young ladies who have passed away through this sickness even though they did treatment and surgery” (P11)

We go through stress when travelling all the way from Bole [where they live] to Tamale

[Where the hospital is located]” (P12)

#### 3.1.2 Emotional Health Experiences

Two themes emerged from the narratives of the FCGs on their emotional health experiences while caring for the cancer patient. The themes are: “Your whole mood changes” and “I am very frustrated”.

*Your whole mood changes*

The emotional swings experienced by the participant varied as the days of caring went by. The responses below are what some participants shared.

“Sometimes you are happy, but when the pain starts, then your whole mood changes …it has really been emotional for me” (P1)

“When I look at her, I'm sad because I know that death is something we cannot avoid, …so I want to help and treat her out. If natural death comes, that one, I can't avoid. Sometimes when I look at her in this situation, I don't even want her to go through that stress because I love my mother, but what can I do?” (P7)

“I got sad when she started complaining about the travelling stress and the money we are using… I wept. Sometimes I come back from work and I see her sad, I get confused” (P12)

“When she is fine, I don't want to be too happy because she will soon become weak, especially after we go for treatment” (P13)

*I am very frustrated*.

Participants also indicated in their narratives that the things they hear about cancer as an impending death affect their emotions, which led to moments of frustration, feelings of guilt when they felt the need to have a break and sleeplessness. Some shared this:

“I'm very frustrated because I have been hearing that cancer kills… that even after treatment, she may not stay long….it's like the treatment is in vain” (P3)

“She has been crying since she was diagnosed, and that frustrates me. If she says we should allow her to die and stop wasting money on her, I get sad” (P5).

“I felt guilty for wanting to take a break; if I was not there for him, who was going to be?” (P7)

“The illness has made me sleepless… Even more so in the night…I get frustrated when I'm not able to provide for my mother and younger brothers” (P10).

“I have been frustrated concerning the finances. It has been a problem for me because I'm my mother's firstborn, and more to the point, my father is no more, so it has been a burden on me” (P11)

#### 3.1.3 Social Health Experiences

Two themes emerged from participants’ narratives on their social health. The themes are: “I don't have time” and “Neglect.”

*I don't have time*

The social health of participants was affected by their roles as caregivers. They indicated that they did not have time to interact with other people as expected, and this affected their social life. Providing long‐term care and having increased responsibility affects FCGs’ social roles, particularly in relation to their educational, professional and personal roles. Participants had this to say:

“For that role, now I've stopped all the building projects and my teaching job because of the illness …so I don't do anything again” (P2)

“I am also having challenges at work. I have been threatened to lose my job if I don't come to work” (P5).

“…the illness has affected my education because I don't have time to study at any time…” (P6)

“I was supposed to get married last year, but because I was always with my mother…, my suitor said I did not have time for him, so he broke up with me” (P13)

*Neglect*

It was deduced from the participants’ experiences that other family members were neglecting them because of the condition of the patient, which required a huge financial commitment. Participants also indicated that they felt people were not treating them rightly because people do not understand the challenges they face as they care for their relatives with cancer. They shared this:

“I usually don't get any support from my family. They will promise, but they won't help” (P8).

“…some relatives do not pick up my calls again…they think I am going to ask them for money.” (P9)

“…some of them don't even care, especially when you are talking about money. The complaints they give to you; you can't think far. …There is no support from the family side” (P11).

“…once in a while, I need to be dependent on others to manage my own family and kids. But sometimes people do not understand the challenges of caring for a sick sister, so they speak ill of me.” (P14)

### 3.2 Coping Strategies Used by FCGs Caring for Adult Patients With Cancer

Participants shared ways they used to cope with their roles as FCGs. Three themes emerged from the narratives of the participants. These themes are: “I know that my God lives”

“We try to adjust” and “Family support”.

#### 3.2.1 Spiritual Health Experiences

One theme emerged from participants’ narratives on their spiritual health. The theme is “I know that my God lives.”

*I know that my God lives*.

Participants relied on God for divine intervention in the process of taking care of the family member with cancer. The following statements were made by the participants regarding what they do for their spiritual health.

“What keeps me going is God's promise in the bible that He is always with us in times of trouble” (P2)

“I know that my God lives…” (P9)

“…I do prayers just on my own with fasting, and we put him [the patient] as a topic, and we pray. We thank God for him and the healing. So, every Monday we do this fasting and prayers and every day we do normal prayers for him” (P7)

“Everything is God's doing, so I always go on my knees and pray to God to take care of us …” (P10)

“…I've been asking for divine help. Every day, that has been my prayer that God sees me through. Sometimes I fast, since this sickness started… It has been God from day one” (P11)

Two other coping strategies identified were within the social health dimension: “We try to adjust” and “Family support.”

*We try to adjust*

Participants indicated that adjustment to the condition is a coping strategy they used. Participants also had to adjust their own routines to be able to take care of their relative.

“I try to cope in the sense that we try to adjust what we used to do first into what is currently happening” (P2)

“ I feel this is normal, so I have adjusted well…I have not sought any divine help” (P6).

“Just dealing with it and moving on with life” (P8)

“I had to balance myself by adjusting to the situation I found myself in” (P12)

*Family support*

Participants indicated that support from family members has been one of the means of coping with their roles as caregivers. Some family members have been supporting both financially and emotionally. Some participants had this to say:

“When we are about to come, I call some of our relatives, they contribute a small amount, and we come. I have support from friends too” (P3).

“It has not been easy coping. I have a lot of support from family and friends. I am living one day at a time” (P5)

“…family members visit and motivate me when I run out of strength; they call to check on our sister and me”. (P8)

“I call close family members about a week before our hospital visit so that they can get money to support me. I show a lot of appreciation and pray for them when they bring me the money for her [the patient] care so that next time, they can get me some more.” (P13)

## 4 Discussion

### 4.1 Psychosocial Experiences of the FCGs

The purpose of this study was to explore the psychosocial health experiences and coping strategies of FCGs of adults with cancer in a Ghanaian teaching hospital. Our findings highlight that FCGs experienced psychological and emotional challenges such as: frustration, sadness, being disturbed, depression and confusion. Several factors could explain these findings, including the diagnosis of cancer, rapid changes in the patient's condition and slow rate of recovery of the patients. Our study shows that caregivers are at increased risk of experiencing psychological distress. They also became frustrated and depressed at some point in time as they viewed the caregiving role as challenging. These are consistent with the findings of a review that suggests that the longer the caregiving role, the greater the tension and emotional burden that could contribute to poor quality of life [30]. In a previous study, caregivers with over 8 years’ experience in the caregiving role described it as frustrating [31]. In a related study, caregivers with poor mental health and social support were likely to have a poor quality of life [32]. This indicates that the findings of the current study are not different from what has been documented in other contexts. Dealing with emotions is one of the unmet needs of the caregiver [19, 33]. An earlier study reported that FCGs were unhappy and felt depressed about the serious side effects of cancer that their patients had to go through, describing them as “horrible” [9]. Our study suggests that caregivers need emotional support with counseling to perform their duties effectively.

Caregivers are often underprepared to perform the many tasks needed to care for their loved ones and often struggle to perform their roles, highlighting the stressful nature of the caregiving role [34]. In Ghana, the caregiving role is a form of ‘on‐the‐job training’ as FCGs get their proficiency over time in the role. As science develops and healthcare systems improve, more complex treatment regimens are increasingly frequent, requiring novel therapies that add to the increasing demands on informal caregivers. The increased day‐to‐day demands on the caregivers intensify their stress. Symptom management and treatment monitoring are some of the complex therapeutic regimens that are provided by caregivers, but they often lack the required information and support to effectively accomplish these tasks [35]. The availability of a well‐designed education on the technicalities of the caregiving role can be a step towards resolving the problem faced by FCGs.

Furthermore, our findings showed that caregivers reported having no time for their usual daily activities, as the caregiving role had taken a larger portion of their time. Some participants had to perform other roles in addition to the caregiving role, while others had to concentrate fully on the caregiving role. A previous study described the caregiving role as a full‐time job that could potentially alter relationships [36]. Parental and spousal roles had been greatly affected by participants who were married and those who were parents. Participants who were students experienced a sudden decline in their academic performance when they took up the caregiving role. A plausible explanation could be that the caregiving role may be so demanding that it makes it almost impossible for them to find additional time to successfully function in other roles.

Empirical evidence suggests that opportunities for flexible work schedules, alternative work arrangements, job‐sharing, and telecommuting could help caregivers manage work‐life while fulfilling caregiving duties. This makes it easy for one to arrange work life such that they can fulfill both their employee and caregiver responsibilities [37]. It is worth mentioning that the participants felt neglected when other family members were not available or close to them. This was due to the patient's condition and the high cost of treatment for cancer, as reported in the narrative. The need for social support is necessary for caregivers. Social isolation is reported to increase the burden on caregivers [38]. A study conducted among caregivers of patients with lung cancer in China recommended the need for increased social support for FCGs in the performance of their roles [39]. This indicates that caregivers require support from other people to reduce the tasks they are challenged with.

## 5 Coping Strategies Adopted by FCGs

Our study also examined the coping strategies used by FCGs caring for adult patients with cancer. The themes identified were “I know that my God lives”, “We try to adjust” and “Family support.”

Spirituality, particularly belief in God and prayer, is viewed as a strategy for maintaining health as well as a mechanism for making a patient compatible with the sickness [40]. This is because faith in God is a vital attribute among most Ghanaians, especially when traditional and orthodox treatments fail; the patient's or caregiver's acceptance of the situation is often regulated by their faith in God. Most Ghanaians hold religious views in high regard, and in the case of tragedy, these values take center stage in their lives [41]. For instance, patients and caregivers want doctors and other authorities to respect their religious views; they also expect them to pay attention to their spiritual needs, and doctors frequently advise patients to develop a relationship with God as part of their recovery therapy [42]. The findings of caregivers exploring their religious and spiritual beliefs are akin to the highly spiritual culture among Ghanaians. A study similar to our study reported that most of the caregivers relied on spiritual leaders and prayers [43]. Our findings also align well with a similar study by Navarta‐Sánchez et al. [44] This affirmed the belief in the supernatural being as a significant aspect of a coping strategy among caregivers in Spain. The above findings reflect the importance that participants in the studies attach to supreme beliefs and the supernatural God. This concept of God in the context of the Ghanaian setting is based on the belief that God has the power to change the condition of every person at the appointed time [41].

Furthermore, our findings suggest that caregivers called on family members for support. This collaborates with the findings of a study conducted in the Volta region of Ghana in which family support was critical for effective coping with the condition [40]. In contrast, FCGs in Hong Kong would rather turn to community services in desperate times for assistance [45]. This result also confirms a similar study by Navarta‐Sánchez et al. [44] where family support was seen as a significant aspect of the coping strategy among caregivers in Spain.

## 6 Conclusions

Caregivers within this study's context had experiences in the four main dimensions of psychosocial health: mental health: “The thinking is many” and “I always feel depressed”; emotional health: “Your whole mood changes” and “I am very frustrated”; social health: “I don't have time” and “Neglect” and spiritual health: “I know that my God lives”. Caregivers adopted two strategies of coping with their roles: “We try to adjust” and “Family support.”

Interventions to improve the psychosocial experiences of FCGs and address the multiple challenges they encounter will promote their health and the outcome of their caregiving roles. Specifically, health education and counseling services should be well incorporated into cancer care to enable caregivers to deal with the psychosocial issues they experience.

Our study highlights that, irrespective of cultural variations, caregivers everywhere experience some measure of burden. Again, interventions to mitigate the impact of caregiving burden must be culturally based and consistent with the global best practices.

## 7 Limitations

While our study broadens an understanding of caregivers’ psychosocial health experiences in Ghana, it has some limitations. First, participants were recruited from only one major health facility in the northern part of Ghana and experiences among caregivers caring for cancer patients may not be a true reflection of the burden of care experienced by caregivers in Ghana. Also, the small sample size makes it difficult to generalize the findings to all caregivers in Ghana. Future studies should consider multiple sites, and a larger sample size could improve representativeness and diversity of this population. Another possible limitation of this study may be related to the background of the people who participated in the study. The majority of them were young and educated. The voices of uneducated caregivers, who often are more present in our setting, have not been heard in this study. The expansion of the sample size may address this limitation. Additionally, this study did not capture the relationship between the patient and the FCG.

## Author Contributions

**Catherine Mensah:** conceptualization, methodology, data curation, investigation, validation, formal analysis, resources, writing – original draft and writing – review and editing. **Salome Amissah‐Essel:** methodology, data curation, validation, formal analysis and writing – review and editing. **Nancy Innocentia Ebu Enyan:** methodology, validation, formal analysis, supervision, resources and writing – review and editing. All authors have read and approved the final version of the manuscript. Nancy Innocentia Ebu Enyan had full access to all of the data in this study and takes complete responsibility for the integrity of the data and the accuracy of the data analysis.

## Funding

The authors received no specific funding for this work.

## Conflicts of Interest

The authors declare no conflicts of interest.

## Transparency Statement

The lead author, Nancy Innocentia Ebu Enyan, affirms that this manuscript is an honest, accurate, and transparent account of the study being reported; that no important aspects of the study have been omitted; and that any discrepancies from the study as planned (and, if relevant, registered) have been explained.

## Data Availability

The datasets used in the current study are available from the corresponding author on reasonable request.
